# Anatomical correlates of apathy and impulsivity co-occurrence in early Parkinson’s disease

**DOI:** 10.1007/s00415-024-12233-3

**Published:** 2024-02-28

**Authors:** Gianpaolo Maggi, Francis Loayza, Carmine Vitale, Gabriella Santangelo, Ignacio Obeso

**Affiliations:** 1 HM-CINAC, Centro Integral de Neurociencias AC. HM Hospitales, Av. Carlos V, 70, Móstoles, 28938 Madrid, Spain; 2 CINC, CSIC, Madrid, Spain; 3 Department of Psychology, University of Campania “Luigi Vanvitelli”, Caserta, Italy; 4 Neurosciences and Bioengineering Laboratory, Faculty of Mechanical and Production Sciences Engineering, Polytechnic University (ESPOL), Guayaquil, Ecuador; 5 Department of Medical, Motor Sciences and Wellness, University “Parthenope”, Naples, Italy; 6 Institute of Diagnosis and Health, IDC-Hermitage Capodimonte, Naples, Italy

**Keywords:** Impulsivity, Impulse control disorders, Apathy, Neuropsychiatry, Addiction, Parkinson’s disease

## Abstract

**Background:**

Although apathy and impulse control disorders (ICDs) are considered to represent opposite extremes of a continuum of motivated behavior (i.e., hypo- and hyperdopaminergic behaviors), they may also co-occur in Parkinson’s disease (PD).

**Objectives:**

We aimed to explore the co-occurrence of ICDs and apathy and its neural correlates analyzing gray matter (GM) changes in early untreated PD patients. Moreover, we aimed to investigate the possible longitudinal relationship between ICDs and apathy and their putative impact on cognition during the first five years of PD.

**Methods:**

We used the Parkinson’s Progression Markers Initiative (PPMI) database to identify the co-occurrence of apathy and ICDs in 423 early drug-naïve PD patients at baseline and at 5-year follow-up. Baseline MRI volumes and gray matter changes were analyzed between groups using voxel-based morphometry. Multi-level models assessed the longitudinal relationship (across five years) between apathy and ICDs and cognitive functioning.

**Results:**

At baseline, co-occurrence of apathy and ICDs was observed in 23 patients (5.4%). This finding was related to anatomical GM reduction along the cortical regions involved in the limbic circuit and cognitive control systems. Longitudinal analyses indicated that apathy and ICDs were related to each other as well as to the combined use of levodopa and dopamine agonists. Worse apathetic and ICDs states were associated with poorer executive functions.

**Conclusions:**

Apathy and ICDs are joint non-exclusive neuropsychiatric disorders also in the early stages of PD and their co-occurrence was associated with GM decrease in several cortical regions of the limbic circuit and cognitive control systems.

**Supplementary Information:**

The online version contains supplementary material available at 10.1007/s00415-024-12233-3.

## Introduction

Neuropsychiatric manifestations of Parkinson’s disease (PD) include a variety of hyperactive and hypoactive behaviors [1]. One of the most prevalent hyper-active behaviors in PD is impulse control disorders (ICDs), characterized by the inability to resist an impulse or desire despite its negative consequences [2]. In contrast, apathy is a frequent hypoactive counterpart of the neurodegenerative PD process, wherein patients experience a general loss of motivation [3]. While both neuropsychiatric disorders seem to be at opposite ends of the motivational continuum, a single patient may encounter them jointly. Hence, hyperactive-impulsive expressions towards certain rewarding stimuli may be counterbalanced by a general hypoactive apathetic state as a general behavioral outcome. Rather than being categorically separated in time, both disorders are theorized as two interrelated entities co-occurring in a continuum [4] in the general population [5] and in neurodegenerative diseases, such as PD [6–9]. However, no study has investigated the underlying neural changes in this association or their pathophysiological relationship in PD.

Aberrant changes along the prefrontal areas and mesocortical pathways are present in both apathy and impulsivity in PD [10, 11], partly explaining the loss of cognitive and motivational outcomes. Importantly, different types of rewards rely on segregated neural networks that account for their differential contribution to both hypo- and hyper-active behaviors such as apathy and impulsivity.

Dysfunctions of the orbitofrontal circuits (OFC) (mainly processing value-based decisions or selection of reward options) have been associated with impulsivity [12], while dysfunctional anterior cingulate cortex (ACC) recruitment (primarily engaged in conflict detection) is often found in patients with both apathy [11, 13] and ICDs [14]. Moreover, several reports have shown morphometric gray matter (GM) abnormalities in the OFC and ACC of PD patients with ICDs (PD + ICD) [15, 16], while others did not confirm these results[17, 18]. Similar conflicting findings have been reported in PD patients with apathy (PD + A) [19] [20, 21] [22].

Therefore, no conclusive remark is currently available on the pathophysiological basis of the co-occurrence of apathy and ICDs in PD. Thus, we exploited the PPMI database to unravel the neural basis underlying the joint emergence of apathy and ICDs (ICD + A) in early untreated PD patients and compare the clinical, demographic, and neurocognitive variables between (i) PD patients with both ICDs and apathy (PD + ICD + A), (ii) PD patients with apathy (PD + A), (iii) PD patients with ICDs (PD + ICD), and (iv) PD patients without apathy and ICDs (PD).

## Methods

### Participants

Data were obtained from the Parkinson’s Progression Markers Initiative (PPMI) database (www.ppmi-info.org/data; accessed on 12th February 2021). A total of 423 PD drug-naïve patients were included, with data obtained yearly at a total of 5 time points since diagnosis. The study population inclusion criteria have been reported elsewhere [23], and all participants were untreated at baseline while they started dopaminergic medication from the 1-year follow-up onwards.

### Clinical, behavioral and neuropsychological assessments

The MDS-Unified Parkinson’s Disease Rating Scale (UPDRS) I to III was used to assess disease severity. The presence and severity of pathological impulsivity (pathological gambling, hypersexuality, compulsive buying, and compulsive eating) and compulsive behaviors (punding, aimless walkabout, and hobbyism) were assessed using the Questionnaire for Impulsive-Compulsive Disorders in Parkinson’s Disease (QUIP) [24]. Subscores for each PD + ICD subtype were also obtained [25]. Apathy was evaluated using item-4 of the UPDRS-I, which assesses motivation and initiation. Patients completed neuropsychological assessment at each annual visit, including evaluation of global cognitive functioning, working memory, processing speed, visuospatial function, language abilities, learning, and long-term verbal memory (neuropsychological evaluation is detailed in Supplementary Material 1). Data from the clinical, behavioral, and neuropsychological evaluations were collected at each visit.

### Statistical analysis

Demographic, clinical, neuropsychiatric, and cognitive variables were compared between PD + A, PD + ICD, PD + ICD + A, and PD groups using Kruskal–Wallis tests. Pairwise comparisons with Bonferroni correction were performed to determine the statistically significant differences. To evaluate the association between the co-occurrence of PD + ICD + A at baseline, we used a Chi-squared (*χ*^2^) test to compare proportions in a two-by-two contingency table.

To establish the longitudinal link between demographic, clinical and neuropsychiatric variables in the PD + A and PD + ICD groups, separate multilevel models (MLM) were used to ascertain the possible predictors of PD + A and PD + ICD across 5 years from diagnosis. Apathy and PD + ICD scores were entered as dependent variables to test time effects (from baseline to 5 years of follow-up). The other variable was then entered as a possible predictor to examine their inter-relationship (no interaction term PD + A × PD + ICD was computed due to mathematical constraints). Demographic, clinical, and neuropsychiatric variables, along with the time of assessment (baseline, 1st, 2nd, 3rd, 4th, and 5th annual follow-up), were entered as predictors. Moreover, we analyzed the longitudinal relationship between PD + A, PD + ICD and cognitive outcomes, considering the interaction term (PD + ICD × PD + A) as a predictor. Full-information maximum-likelihood parameter estimation was used to account for missing data in MLM analyses. All analyses were performed using IBM SPSS Statistics Version 24.

### Neuroimaging data analysis

T1-weighted anatomical MRI images at baseline were downloaded from the PPMI database for each patient group. Thus, from the total number of images, 48 corresponded to PD + A, 56 to PD + ICD, 21 to PD + ICD + A, and 245 to PD.

To account for morphometric changes between groups (i.e. Voxel-based morphometry, VBM analysis), the GM images were normalized to the MNI template using Dartel. Next, an 8 × 8 × 8 Gaussian FWHM smooth kernel was applied. Due to the unbalanced sample size between groups, nonparametric testing (SnPM using 5000 permutations without variance smoothing) was used to compare voxel-wise differences in GM brain anatomy focal differences. Analyses were performed using ANOVA with covariates to adjust for sex and unequal variances. Post-hoc comparisons were performed using a nonparametric two-sample *t*-test: PD + A, PD + ICD, and PD + ICD + A were compared with the PD group. Additional comparisons were performed to test for differences between PD groups. For all contrasts, between-group differences were assessed using FWE at *p* = 0.05, corrected for multiple comparisons. Standard procedures for VBM analysis were used, including global normalization using the total intracranial volume, to correct the variation in the regional volume due to different brain sizes. Imaging data was analyzed with SPM12 for pre-processing and SnPM13 for non-parametric statistics [26] in Matlab R2016b.

## Results

### Co-occurrence of apathy and ICDs in early drug-naïve PD patients

**Fig. 1:**
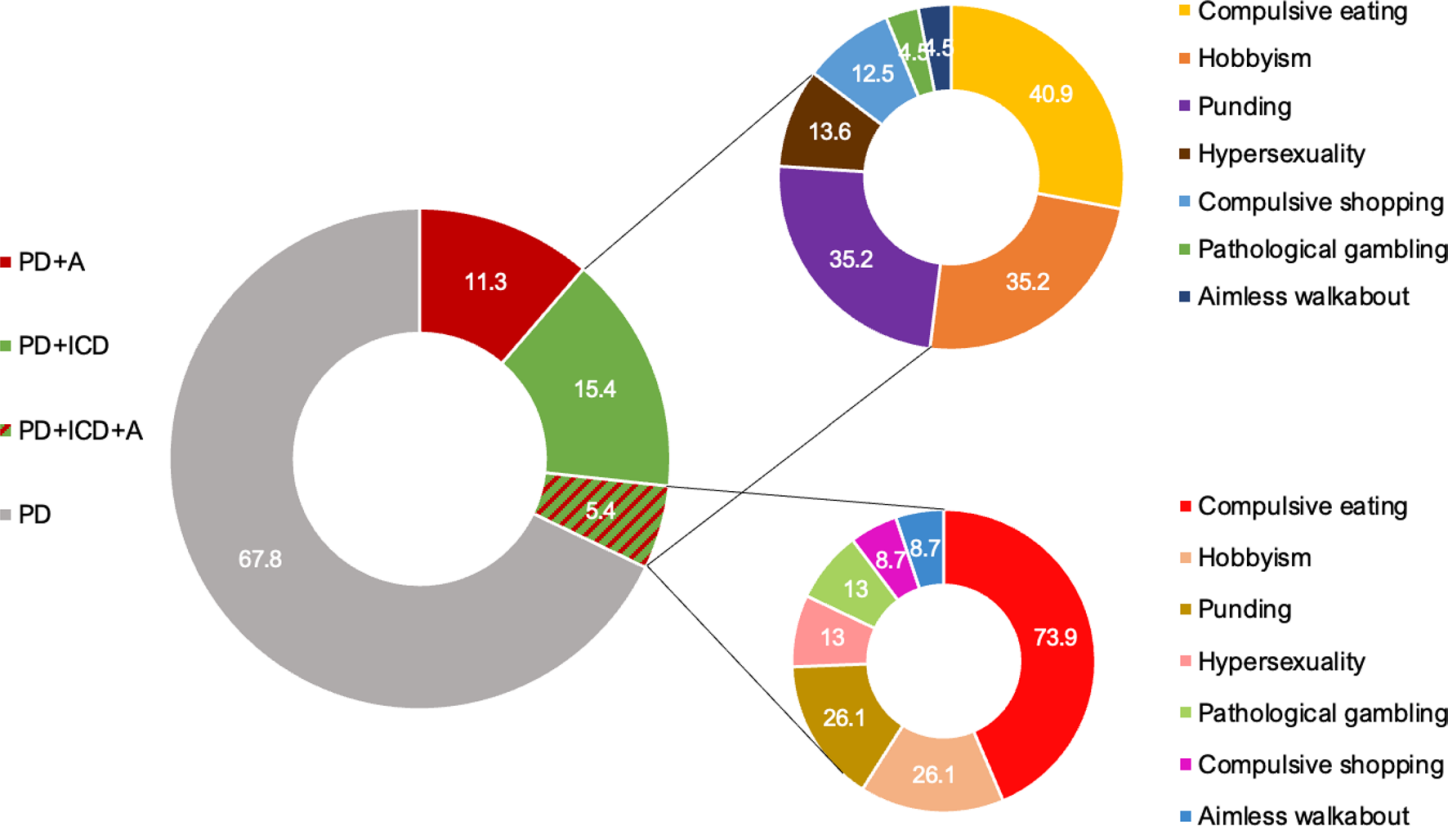
Percentages of groups distribution according to the presence of apathy (PD + A), PD + ICD (PD + ICD), patients with the co-occurrence of apathy and PD + ICD (PD + ICD + A) and PD without ICD and apathy (PD). Frequencies for each PD + ICD sub-type were described for all the patients reporting PD + ICD and for the PD + ICD + A group. Note that the sum of the percentages of specific ICDs may exceed 100% due to comorbidities

At baseline assessment, 287 PD patients (67.8%) reported neither apathy nor ICDs symptoms. Meanwhile, 48 patients (11.3%) experienced apathy and 65 (15.4%) experienced ICDs. The co-occurrence of ICD + A was observed in 23 patients (5.4%) (Fig. 1). Interestingly, the total number of apathetic patients, considering apathy alone or in combination with ICD, was 71, while the total number of patients reporting ICDs alone or in combination with apathy was 88. Of the 88 PD + ICD patients, compulsive eating was the most frequent ICDs subtype since it was reported by 36 patients (40.9%); 21 PD + ICD patients (35.2%) reported hobbyism, 21 patients (35.2%) reported punding, and 12 (13.6%) and 11 (12.5%) PD + ICD patients complained of hypersexuality and compulsive buying, respectively. Finally, pathological gambling and aimless walkabout were observed in 4 patients (4.5%) (Fig. 1).

Chi-squared tests of independence showed a significant relationship between the co-occurrence of ICD + A in early drug-naïve PD patients (*df* = 1; *χ*^*2*^ = 6.957; *p* = 0.008). Particularly, we observed more PD and PD + ICD + A than statistically expected (expected count: 278.8 and 14.8, respectively), whereas PD patients with only one disorder were less than expected in our sample (expected count: PD + ICD = 73.2 and PD + A = 56.2). The longitudinal evolution of PD + ICD + A was heterogenous. Of the 23 patients categorized as PD + ICD + A at baseline, 17 had completed the 5-year follow-up evaluation. Of these, six patients reported PD + ICD + A also at follow-up, while 8 patients presented one of both neuropsychiatric conditions (two patients continued as PD + ICD, six patients were PD + A), whereas 3 PD patients did not report neither ICDs nor apathy.

### Baseline clinical and demographic features

To further categorize the demographic and clinical factors associated with the co-occurrence of ICD + A, we compared the motor and non-motor scales between the groups. No significant differences on demographic variables were found between the groups except for the H&Y score, which was significantly higher in the ICD + A group (*p* = 0.023) (Table 1). Moreover, the UPDRS-I scores on neuropsychiatric state revealed that PD obtained the lowest scores compared to other three groups, followed by PD + ICD group, whose scores were significantly lower than those of PD + A and PD + ICD + A (Table 1). PD + A reported greater general cognitive dysfunction than PD group (*p* = 0.001), while PD + A and PD + ICD reported more hallucinations than PD group (Table 1). As for the UPDRS-II, PD + ICD + A reported more difficulties in daily activities than PD + ICD (*p* = 0.019) and PD (*p* < 0.001) groups as well as PD + A compared to PD group (*p* < 0.001).

**Table 1:** Comparison of neuropsychiatric subgroups on socio-demographical, clinical, neuropsychiatric and neuropsychological variables

	PD (*n* = 287) *A*	PD + A (*n* = 48) *B*	PD + ICD (*n* = 65) *C*	PD + ICD + A (*n* = 23) *D*		
	Mean ± SD	Mean ± SD	Mean ± SD	Mean ± SD	*χ*^2^	*p*
Age	61.45 ± 9.51	60.98 ± 9.81	60.23 ± 10.45	60.65 ± 10.27	0.608	0.894
Educational level (ys)	15.70 ± 3.05	15.06 ± 2.82	15.37 ± 2.75	15.30 ± 2.90	1.966	0.580
Gender	188 M/99 F	33 M/15 F	41 M/24 F	15 M/8 F	0.394	0.941
Clinical variables						
Disease duration (m)	25.96 ± 22.00	23.18 ± 21.28	24.09 ± 21.18	30.67 ± 30.00	0.424	0.935
H&Y	1.56 ± 0.51	1.67 ± 0.48	1.43 ± 0.50	1.78 ± 0.42	11.018	**0.012** **D > C**
UPDRS-I score	4.43 ± 3.38	9.17 ± 4.52	6.20 ± 3.49	10.65 ± 3.90	95.832	** < 0.001** **B, C, D > A** **B, D > C**
UPDRS-I Cognition	0.22 ± 0.47	0.50 ± 0.58	0.37 ± 0.52	0.48 ± 0.66	21.794	** < 0.001** **B > A**
UPDRS-I Hallucinations	0.01 ± 0.10	0.08 ± 0.28	0.08 ± 0.27	0.04 ± 0.21	13.171	**0.004** **B > A, C > A**
UPDRS-I Depression	0.16 ± 0.40	0.65 ± 0.84	0.32 ± 0.50	0.78 ± 0.79	46.583	** < 0.001** **B > A, D > A, D > C**
UPDRS-I Anxiety	0.34 ± 0.54	0.60 ± 0.89	0.51 ± 0.64	0.83 ± 0.78	16.396	**0.001** **D > A**
UPDRS-I Apathy	0.00 ± 0.00	1.17 ± 0.52	0.00 ± 0.00	1.22 ± 0.42	420.157	** < 0.001** **B > A, B > C,** **D > A, D > C**
UPDRS-I DDS	0.02 ± 0.15	0.02 ± 0.14	0.05 ± 0.21	0.04 ± 0.21	3.181	0.365
UPDRS-I Fatigue	0.56 ± 0.73	1.04 ± 0.92	0.53 ± 0.62	1.35 ± 0.98	32.482	** < 0.001** **B > A, B > C,** **D > A, D > C**
UPDRS-II score	5.28 ± 3.94	8.17 ± 4.61	5.80 ± 3.72	9.26 ± 4.57	32.387	** < 0.001** **B > A, D > A, D > C**
UPDRS-III score	20.95 ± 9.31	22.90 ± 7.83	18.92 ± 8.00	21.48 ± 6.31	7.657	0.054
Rigidity	3.75 ± 2.71	4.25 ± 2.51	3.46 ± 2.65	4.26 ± 2.07	5.888	0.117
Tremor	4.40 ± 3.19	4.15 ± 3.37	4.48 ± 2.94	3.74 ± 2.73	1.450	0.694
UPDRS total score	30.66 ± 13.05	40.23 ± 12.74	30.80 ± 11.26	41.39 ± 10.52	35.835	** < 0.001** **B > A, B > C,** **D > A, D > C**
M-S&E ADL score	93.96 ± 6.04	91.15 ± 5.48	93.69 ± 5.54	93.04 ± 5.16	7.376	0.061
ESS	5.62 ± 3.45	5.56 ± 3.30	6.15 ± 3.49	7.61 ± 3.42	8.343	**0.039** **D > A**
RBDQ	3.93 ± 2.65	4.40 ± 2.43	4.64 ± 2.80	4.48 ± 3.19	5.636	0.131
SCOPA-AUT	8.23 ± 5.64	11.19 ± 4.84	11.82 ± 6.73	15.09 ± 7.68	43.505	** < 0.001** **B > A, C > A, D > A**
UPSIT	22.61 ± 8.37	22.13 ± 8.33	21.42 ± 7.40	22.22 ± 8.83	1.301	0.729
Neuropsychiatric Variables						
GDS	1.84 ± 2.09	3.65 ± 3.19	2.74 ± 2.32	4.39 ± 2.90	41.507	** < 0.001** **B > A, D > A**
STAI	61.83 ± 17.02	73.90 ± 20.65	70.95 ± 17.29	75.65 ± 19.01	36.279	** < 0.001** **B > A, C > A, D > A**
QUIP	0.00 ± 0.00	0.00 ± 0.00	1.25 ± 0.50	1.70 ± 0.93	416.679	** < 0.001** **C > A, C > B, D > A, D > B**
Neuropsychological variables						
MoCA	27.22 ± 2.34	27.04 ± 2.08	26.69 ± 2.49	27.35 ± 1.90	3.395	0.335
SDMT	41.02 ± 9.21	41.29 ± 9.42	41.60 ± 12.21	41.74 ± 9.31	0.388	0.943
LNS	10.60 ± 2.60	10.48 ± 2.47	10.35 ± 3.11	11.22 ± 2.41	1.508	0.680
HVLT Immediate recall	24.63 ± 4.99	23–94 ± 4.72	23.97 ± 5.30	24.57 ± 4.61	1.979	0.577
HVLT Delayed recall	8.38 ± 2.51	8.40 ± 2.16	8.11 ± 2.82	8.74 ± 2.40	0.970	0.808
Semantic fluency	48.77 ± 11.42	47.83 ± 13.81	49.22 ± 11.28	47.61 ± 10.92	1.251	0.741
BJLOT	12.77 ± 2.15	12.90 ± 2.11	12.57 ± 2.28	13.13 ± 1.36	0.806	0.848

*PD* Parkinson’s Disease, *PD + A* *PD* patients with apathy, *PD + ICD* *PD* patients with impulse control disorders, *PD + ICD + A* PD patients with both apathy and impulse control disorders, *H&Y* Hoehn and Yahr staging system, *UPDRS* Unified Parkinson’s Disease Rating Scale, *M-S&E ADL* Modified Schwab and England Activities of Daily Living, *GDS* Geriatric Depression Scale, *STAI* State-Trait Anxiety Inventory, *QUIP* Questionnaire for Impulsive-Compulsive Disorders in Parkinson's Disease, *ESS* Epworth Sleepiness Scale, *RBDQ* REM Behavior Disorder Questionnaire, *SCOPA-AUT* SCales for Outcomes in PArkinson's disease – Autonomic Dysfuntions, *UPSIT* University of Pennsylvania Smell Identification Test, *MoCA* Montreal Cognitive Assessment, *SDMT* Symbol Digit Modalities Test, *LNS* Letter Number Sequencing, *HVLT* Hopkins Verbal Learning Test, *BJLOT* Benton Judgement of Line Orientation. Statistically significant values are reported in bold

No significant difference was observed on the UPDRS-III score between groups whereas motor subtypes significantly differed across the groups (*df* = 3; *χ*^*2*^ = 9.958; *p* = 0.019), with the PD + ICD + A group characterized by worse postural instability/gait disorder subtype and less tremor-dominant features.

Neuropsychiatric variables showed differential scores for depressive and anxiety symptoms, with the control group showing less severity of depression and anxiety than the other groups (Table 1). As expected, PD + ICD + A and PD + ICD reported significantly higher QUIP scores than PD + A and PD (*p* < 0.001). Regarding non-motor and autonomic symptoms, the PD group reported less autonomic dysfunctions than the other groups (Table 1) whereas PD + ICD + A reported more excessive daytime sleepiness than the PD group (*p* = 0.034). No significant difference between groups emerged on cognitive variables (Table 1).

### Baseline neuroimaging findings

**Fig. 2:**
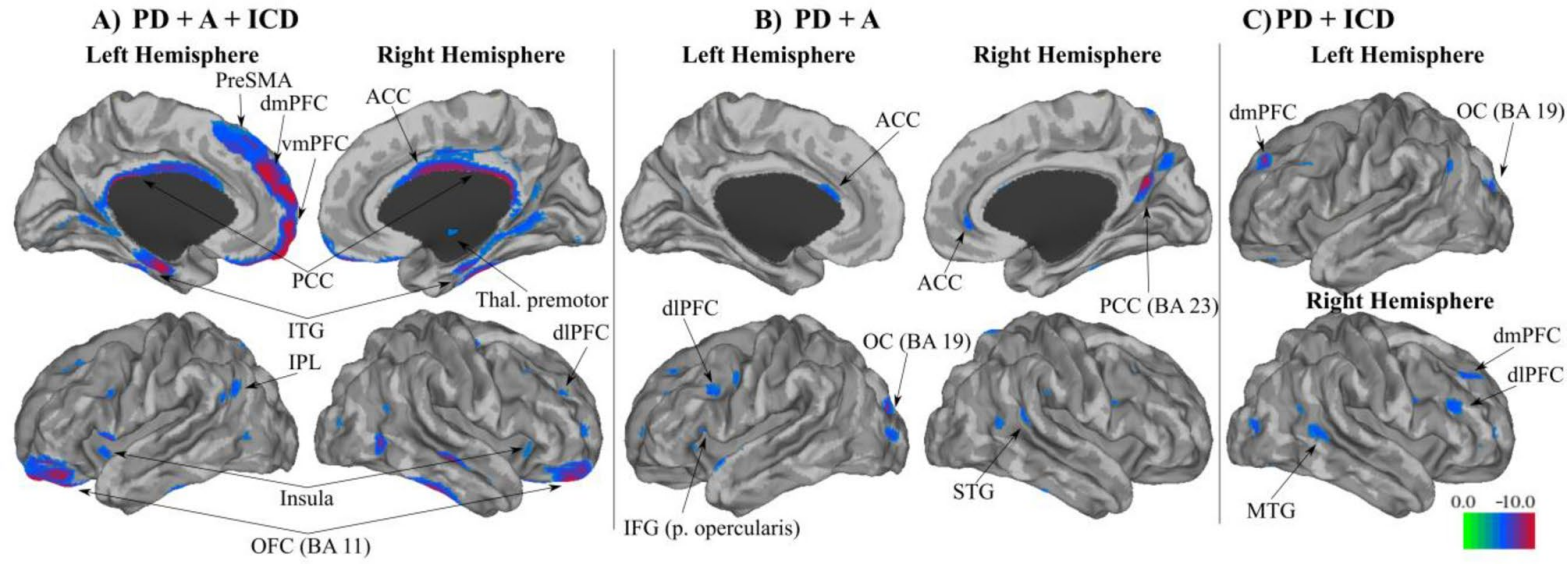
Whole-brain focal differences in gray matter for the co-occurrence of PD + ICD + A. Imaging results reveal significantly reduced GM density for: **A** the co-occurrence of apathy and ICD (PD + ICD + A), **B** PD + A, and, **C** PD + ICD compared to baseline PD without ICD and apathy (PD). Each group was compared independently through nonparametric random effects analysis as shown in the three panels. No significant increases in GM density were found for the PD neuropsychiatric groups compared to PD; FWE corrected at *p* = 0.05

**Fig. 3:**
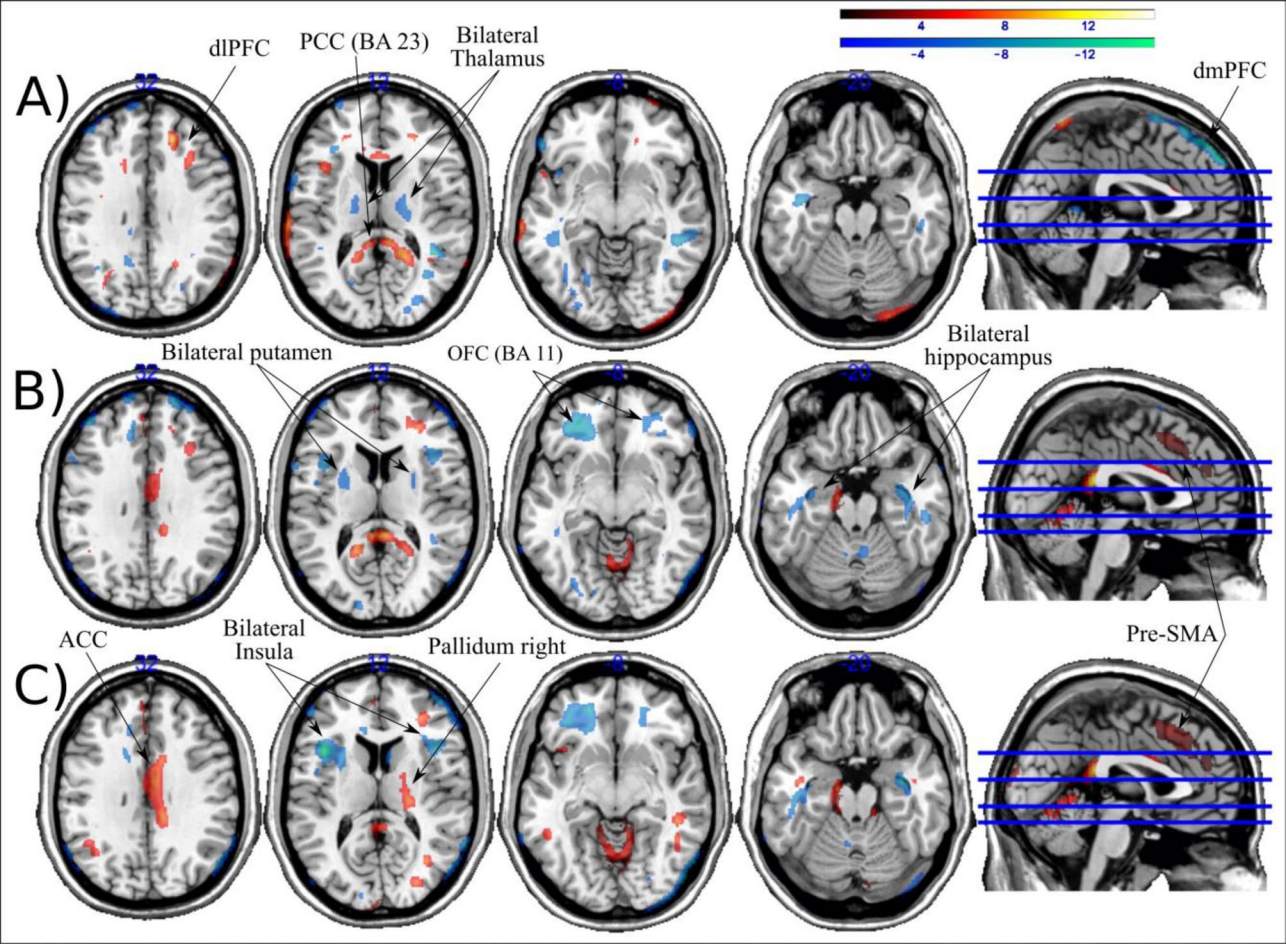
Post-hoc comparisons reveal significant changes in cortical areas within the neuropsychiatric PD groups. **A** Comparison between PD + ICD vs PD + A; **B** Comparisons between PD + ICD vs PD + ICD + A; **C** Comparisons between PD + A vs PD + ICD + A. All contrast were evaluated using FWE corrected at *p* < 0.05 and extent threshold of 100 voxels. Increased GM density in hot and reduced in cold colors

Despite the volumetric differences in GM, WM, and CSF across subjects, we did not find any significant differences between groups for each tissue class. As expected, larger GM tissue concentrations were found in the PD group than in the other PD neuropsychiatric groups (Fig. 2; Table 2). Importantly, patients experiencing PD + ICD + A exhibited reduced GM density in the prefrontal areas, including ACC, OFC, dorsomedial prefrontal cortex (dmPFC), ventromedial prefrontal cortex (vmPFC) and pre-SMA (Fig. 2; Table 2) compared to PD. As for the comparisons performed within the PD neuropsychiatric groups (Fig. 3), PD + ICD (compared to PD + A patients) showed increased cortical hubs, mainly in the right dlPFC and PCC and reduced GM density in the bilateral thalamus and dmPFC. In addition, PD + ICD showed (compared to PD + A + ICD patients) increased ACC, right dlPFC and pre-SMA; and reduced GM density in bilateral regions of OFC, putamen, insula and hippocampus. Finally, PD + A (compared to PD + ICD + A patients) exhibited increased GM density in ACC, pre-SMA, right pallidum; and decreased in bilateral insula and hippocampus (Fig. 3). These anatomical variations in executive control and limbic cortical regions may explain the co-occurrence of apathy and ICD in PD.

**Table 2:** Gray Matter reductions in tissue concentration per group (compared to PD)

Group	Anatomical Region	BA	Side	*T*	*X*	*Y*	*Z*
PD + ICD + A	Ventro-medial PFC	11/10	L	13.46	– 3	59	– 26
	Orbito-frontal cortex	11	L/R	11.2	– 28	51	– 17
	Inferior temporal gyrus	20	L/R	9.55	35	– 22	– 33
	Pre-SMA	6	L	5.55	– 2	16	54
	ACC	33	L/R	8.98	5	– 6	30
	Posterior cingulate	23/33	R	9.11	6	– 34	25
	Dorso-medial PFC	32	L	9.82	– 2	48	28
	Insula	48	L/R	8.23	– 50	14	– 1
	Dorso-lateral PFC	47/44	R	8.8	29	42	9
	Infero-parietal cortex	39	L	9.1	– 39	– 52	27
	Thalamus premotor	–	R	4.97	23	– 15	15
	LVIIa Cerebellum (Crus 1)	–	L/R	7.77	– 29	– 62	– 41
PD + A	Posterior Cingulate	23	R	8.24	21	– 59	29
	Infero-frontal gyrus (p. Opercularis)	48	L	8.29	– 41	17	12
	ACC	24	L	4.72	0	24	18
		10	R	5.45	1	59	4
	Occipital Cortex	18	L	7.93	– 23	– 85	5
	Superior Temporal G	37	R	7.18	45	– 53	6
	LVIIa Cerebellum (Crus 1)	–	R	7.93	40	– 85	– 36
	Dorsal Dentate Nucleus	–	L	7.09	– 20	– 56	– 33
PD + ICD	Dorso-medial PFC	46	L/R	7.48	– 23	41	23
		48	R	5.69	42	33	21
	Middle Temporal Gyrus	21	R	5.58	55	– 44	2
	Occipital Cortex	19	R/L	10.15	33	– 76	8
	Thalamus motor	–	R	6.37	21	– 18	6
	Cerebellum Lobule VIIa crusI	–	R	7.85	53	– 67	– 43
	Cerebellum (VIII)	–	L	10.85	– 21	– 65	42

*PFC* prefrontal cortex, *ACC* anterior cingulate cortex, *pre-SMA* pre-supplementary motor area, *PD* Parkinson’s disease, *PD + ICD + A* PD patients with impulse control disorders and apathy, *PD + ICD* PD patients with impulse control disorders, *PD + A* PD patients with apathy

### Longitudinal relationship between PD + A and PD + ICD with clinical, neuropsychiatric and socio-demographic variables over the course of the disease

As expected, the MLM showed that PD + ICD was related to the use of levodopa or dopamine agonists (DA) alone, the use of levodopa and DA alone and in combination with other medications, younger age and greater levels of apathy (Table 3). Time was not associated with the development of PD + ICD.

**Table 3:** Multilevel models predicting the development of ICDs and apathy from clinical, neuropsychiatric and socio-demographic variables

Predictors	ICDs			Apathy		
	Beta	SE	*p*	Beta	SE	*p*
Age	– 0.009	0.004	**0.026**	– 0.003	0.003	0.288
Sex	– 0.043	0.050	0.383	– 0.040	0.039	0.298
Level of education	0.010	0.008	0.211	– 0.003	0.006	0.608
Type of onset	0.117	0.086	0.175	0.096	0.067	0.151
H&Y	– 0.013	0.035	0.710	0.077	0.030	**0.010**
UPDRS-III	– 0.000	0.002	0.985	0.005	0.001	** < 0.001**
LEDD	– 0.000	0.000	0.865	– 0.000	0.000	0.763
Levodopa	– 0.033	0.058	0.565	0.140	0.049	**0.005**
DAs	0.074	0.059	0.212	– 0.079	0.051	0.120
Others	– 0.036	0.055	0.514	0.002	0.047	0.970
Levodopa + Other	0.056	0.076	0.463	0.112	0.064	0.083
Levodopa + DAs	0.302	0.074	** < 0.001**	– 0.013	0.063	0.834
DAs + Other	– 0.019	0.066	0.770	0.056	0.057	0.324
Levodopa + DAs + Other	0.219	0.085	**0.010**	– 0.045	0.073	0.533
Depression	0.043	0.026	0.092	0.349	0.020	** < 0.001**
Anxiety	– 0.004	0.023	0.853	0.110	0.019	** < 0.001**
Apathy	0.067	0.025	**0.008**	–	–	** – **
ICDs	–	–	** – **	0.050	0.018	**0.007**
Time	0.013	0.012	0.270	0.010	0.010	0.321

*ICDs* Impulse control disorders, *H&Y* Hoehn and Yahr staging system, *UPDRS* Unified Parkinson’s Disease Rating Scale, *LEDD* Levodopa Equivalent Daily Dose, *DA* Dopamine Agonists. Statistically significant values are reported in bold

Moreover, MLM analysis showed that more severe apathy was related to the use of levodopa alone, worse motor condition (UPDRS-III), higher H&Y, depression and anxiety scores, and ICDs (Table 3). To examine which subtype of ICDs was mostly related to apathy, an identical MLM analysis was carried out by entering each ICDs subtype score. We found that greater apathy levels were related to the use of levodopa alone as medication, worse motor symptoms (UPDRS-III), higher H&Y scores, more severe anxiety and depression symptoms, and compulsive eating and aimless wandering (Supplementary Material 2). Therefore, MLM analyses indicated that A and ICDs were related to each other during the first five years after diagnosis. Importantly, the development of A was predicted by two ICDs sub-domains (compulsive eating and aimless wandering).

### Longitudinal relationship between co-occurrence of PD + A and PD + ICD over the course of the disease

MLM analyses showed that poorer performance on cognitive flexibility (measured by Letter Number Sequencing, LNS) was linked to advanced age, fewer years of education, time of evaluation (i.e., performance worsened over time), and more severe A and ICDs (Supplementary Material 3). To further explore which ICDs subtype was mostly related to performance on LNS, the same MLM analysis was carried out by entering each ICDs subtype QUIP score. We found that lower scores on LNS were related to advanced age, lower educational level, time of evaluation, and compulsive buying and punding (Supplementary Material 3).

Moreover, worse executive functions on speed processing and working memory (SDMT) were associated with advanced age, lower educational level, male sex, higher UPDRS-III score, time of evaluation, and more severe anxiety and apathy (Supplementary Material 4). No effect of apathy and/or ICDs emerged for the other predictors (see Supplementary Material 4).

## Discussion

In the present study, we observed co-occurrence of apathy and ICDs in 5.4% of early drug-naïve PD patients. Meanwhile, 48 patients (11.3%) experienced apathy and 65 (15.4%) experienced ICDs. Interestingly, compulsive eating and aimless wandering emerged as the ICDs subtypes most closely associated with A. PD + ICD + A patients showed significantly worse H&Y and non-motor symptoms as well as higher UPDRS-I and UPDRS-II scores than other groups.

The anatomical changes associated with the co-occurrence of ICDs and apathy showed GM reductions in the ventral and lateral prefrontal areas, including the ACC, pre-SMA, DLPFC, OFC, and insula, compared with PD control patients. We also found subcortical reductions in the cerebellum and thalamus in patients with co-occurrence. However, no significant differences were observed in striatal regions. Finally, worse executive control was predictive in the long-term by the presence of PD + ICD + A, specifically linked to compulsive buying and punding subtypes of PD + ICD.

Our prevalence rate of PD + ICD + A (5.4%) was lower than that reported by Scott and colleagues [9] (17%), probably because of substantial differences in the enrolled samples. Indeed, we explored this co-occurrence in early untreated PD patients, whereas Scott and colleagues [9] enrolled patients in a more advanced PD stage (approximately 10 years of disease duration) under dopaminergic medication. These findings suggest that although dopaminergic replacement treatments (DRT) significantly contribute to apathy and ICDs genesis, this does not fully explain their co-occurrence in PD.

Moreover, the PD + ICD + A group had a more advanced PD stage and more severe non-motor symptoms than the other groups, in line with the results presented by Scott and colleagues suggesting that the co-occurrence of apathy and ICDs in PD may represent a malignant clinical phenotype characterized by disabling manifestations and faster progression in overall prognosis.

We found possible neurobiological variations to explain the co-occurrence of PD + ICD + A, involving GM reductions in ventromedial and lateral prefrontal cortex. Specifically, the novelty of our findings lies in the cortical reductions present in the co-occurrence group along the OFC, pre-SMA, vmPFC, dmPFC, and insula. Similar reductions in the OFC have been reported in PD + ICD as a potential explanation for emotional uncontrolled responses [15]. The implication of the OFC in the expression of PD + ICD + A co-occurrence comes as no surprise, given the role of the OFC in mediating motivated behaviors [27] or selection of the most convenient reward action [28]. Thus, these functions may be diluted in the co-occurrence of most (but not all) rewarding stimuli to explain apathy. The affective-limbic areas found to be decreased in the co-occurrence imply the ACC and insula, possibly mediating the downregulation of most motivated changes in both syndromes. However, patients with PD + ICD + A may still be interested in some motivated behaviors showing excessive uncontrolled actions and resulting in PD + ICD. In contrast, the underlying changes mediating impulsive-like behavior can be attributed to anatomical reductions in cognitive control areas found in the current study, including the medial prefrontal cortex and pre-SMA. Indeed, abnormal activity of the dorsal medial PFC and pre-SMA areas has been reported in PD + ICD [29], associated with abnormal control in PD + ICD due to its role in executive control and response inhibition [30]. An alternative non-exclusive view would entail that the reduced GM density in the pre-SMA participate in the co-occurrence of dysfunctional changes in various behavioral roles mediated via limbic structures [31]. On the one hand, pre-SMA reductions mark the inability to spontaneously self-activate without external stimulation in apathetic patients [32], which would entail long-term hypoactive behavior to general contextual stimulation. Indeed, previous reports suggest that higher levels of apathy are associated with reduced functional recruitment of the SMA complex (including the pre-SMA) and ACC in apathetic PD patients [11]. On the other hand, pre-SMA reductions lead to failed control over specific reward items that are of great interest to patients. Therefore, we envisage the pre-SMA as a critical hub mediating PD + ICD + A by favoring hypo-active apathetic behaviors most of the time, while impulsivity on some occasions in response to specific rewards.

Similar changes were observed in the cortical profile of the DLPFC in PD + ICD [10] and PD + A [11] as part of the executive control prefrontal network. We also replicated ACC reductions in PD + A, a neural marker of reduction in reward search and production of apathy in PD [11]. The involvement of OFC and ACC in PD + ICDs has shown lower GM volumes [15], but also increased GM density in other reports [16, 33]. This inconsistency may be driven by the sample characteristics of different studies, including different PD + ICD subtypes or other methodological constraints such as power or sample size. Nonetheless, reductions in several cortical areas, including the pre-SMA, vmPFC, dmPFC, and insula in PD + ICD + A are specifically and mostly exclusively implicated in co-occurrence.

Along with the above prefrontal changes seen in PD + ICD + A, tonic dopamine release can lead to critical modulations in the reward and valuation networks. Within the same PD + ICD + A patient, it may be plausible to expect an opposing effect of dopaminergic modulation over the reward and control system in response to contextual stimuli. While imaging analysis was performed on patients at baseline without medication and no functional measurements, the anatomical variations behind PD + ICD + A were independent of levodopa or dopamine agonist intake. Therefore, consistent with the “overdose hypothesis” [34], decreased or enhanced dopamine release in the mesocorticolimbic route will force patients to stick to either hypo- or hypersensitivity, respectively, in response to rewards together with lost control over behavior. In line with the dopamine view, evidence on healthy samples indicates that different reward subtypes count with segregated cortico-subcortical networks [28]. A possible mechanistic account of PD + ICD + A co-occurrence may be the excessive recruitment of a specific reward circuitry to guide impulsive actions while simultaneously reducing the presence of all other limbic pathways to explain the loss of general motivation in the same patient. Hence, co-occurrence will be guided by a differential contribution of specialized prefrontal networks that are unbalanced in hypo- and hyper-active behaviors relative to apathy and impulsivity. Although neurodegenerative processes and dopaminergic replacement treatments play significant roles in apathy and ICDs genesis, their etiology may be considered a multifactorial process. Such a multifactorial view fits with the notion of different susceptibility factors in both conditions, such as genetic and personality traits, cognitive dysfunctions, and sociocultural or contextual factors [35].

The impact of abnormal dopamine signals in PD + ICD + A may be relevant for PD + ICD [36] but less clear for PD + A [37]. Dopamine levels in the striatum seem to be unimpaired in PD + A[38], but also the contrary has been reported[37], whereby recent suggestions indicate alternative neurotransmitters such as serotonin [39] or noradrenaline [40] may mediate apathy. An alternative view relates to changes in the functional properties of dopamine in the presence of particular rewarding stimuli. Dopamine would modulate apathy by levering alertness or interest over external rewarding stimuli when hypoactive states dominate behavior, together with sustained changes in a large affective-limbic network [37, 41]. Consistent with the “overdose hypothesis”, dopaminergic treatment plays an uneven role in cortical-subcortical representations by impairing cognitive control functions in hyperactive behaviors (i.e., PD + ICD), explained by the pre-SMA anatomical changes observed in PD + ICD + A co-occurrence. Indeed, the PD + ICD group had larger ACC, right dlPFC, and pre-SMA than the co-occurrence group. Taken together, our anatomical findings indicate that PD + ICD + A patients experience apathetic symptoms related to regions that, together with dopamine, reduce their contribution to self-activation and obtain pleasure from ordinary activities. This idea seems to be supported by our analyses of behavioral data, revealing that the presence of apathetic symptoms in early PD was related to aimless wandering, repetitive behavior without goal-directed aims, and compulsive eating, a reward-seeking behavior. Indeed, binge eating is frequently present in PD and is perfectly compatible with a severe apathetic state (e.g., lying down in the sofa while eating without control). The combination of binge eating and apathy may not require great effort to be conducted in synchrony, boosted by the consumption of high-fat and high-sugar foods that activate reward-related neural circuitry [42].

In the long-term, we found that early signs of PD + ICD and PD + A (separately) increased dysfunctions of the executive system. Consistent with prior findings revealing lower scores on executive tests in apathetic [43, 44] and PD + ICD patients [35, 45] compared to PD controls, our findings add to the literature on the long-term risks of neuropsychiatric signs on executive functioning in PD. Moreover, we found poorer performance on LNS, a task evaluating flexibility and working memory, to be related to punding and compulsive buying. Punding is defined as an intense fascination with excessive, repetitive, and habit-oriented behaviors (e.g., cleaning, examining objects, or arranging) [46]. Meanwhile, compulsive buying is characterized by an uncontrolled capacity to refrain from shopping and buying behavior, leading to adverse psychological, social, and financial consequences. Purchase consummatory behavior assumes a situation in which economic decisions are made, requiring the retrieval and organization of salient cognitive and affective information. Therefore, flexibility and working memory dysfunctions interfere with adapted behavior based on patients’ needs, where a lack of flexibility anticipates affective reactions and their consequences (rewards and punishments) during decision-making [47]. Although the neuropsychological features of punding have been underexplored, flexibility and working memory dysfunctions seem to play major roles [46].

Considering that neuropsychiatric illnesses are risk markers for subsequent cognitive decline [43, 44], early identification of these conditions in neurological populations may allow the prompt implementation of strategies against later cognitive decline and dementia.

### Limitations

Our study has several limitations that need to be addressed. First, while current apathy measures in PD include the Apathy Evaluation Scale and other questionnaires, the current PPMI apathy scale is item-4 of the UPDRS-I. Although the UPDRS-I single item demonstrates adequate sensitivity and specificity and may be employed for rapid screening in regular clinical practice [9, 48], it does not represent the gold standard for apathy assessment and might limit our classification in terms of severity and apathy subtypes in the study of co-occurrence. Therefore, future studies to test apathy and ICDs co-occurrence should employ validated scales to evaluate apathetic symptoms in PD (such as the Apathy Evaluation Scale and Dimensional Apathy Scale) to better characterize the possible relationship with ICDs and other neuropsychiatric symptoms. Second, longitudinal clinical progression of PD + ICD + A co-occurrence was not possible due to the limited number of patients included in this group (*n* = 23). Hence, we employed the entire database to explore their interactions with variables of interest. Third, the groups were largely unbalanced (range: 23–287 patients), inducing unequal variance between them. To overcome this unplanned issue, we used non-parametric tests for analysis and results. Lastly, the assessment of neuropsychiatric disorders using self-report scales such as the QUIP may be misleading because patients might be unaware of their disorders or minimize the presence of such disturbances due to sociocultural factors or social embarrassment. Therefore, it is advisable to assess these disturbances using ad hoc validated scales and combining their self-report evaluations with their caregivers’ reports.

In conclusion, our findings provide further evidence on the need for a conceptualization of PD + A and PD + ICDs that overcomes the limited view of these two disorders as opposing extremes of motivated behavior. More specifically, PD + ICD + A co-occurrence represents a clinical PD phenotype characterized by more disabling motor and non-motor manifestations and a greater extent of degeneration involving several areas of the limbic and cognitive control systems at an early stage in PD evolution, which may also predict executive dysfunctions within 5 years from the diagnosis.

### Supplementary Information

Below is the link to the electronic supplementary material.

Supplementary file1 (DOCX 14 KB)

Supplementary file2 (DOCX 15 KB)

Supplementary file3 (DOCX 19 KB)

Supplementary file4 (DOCX 22 KB)

## Data Availability

Datasets associated with the present study are available upon reasonable request of interested researchers.
