# Supplementary material for: Anatomical correlates of apathy and impulsivity co-occurrence in early Parkinson’s disease
Source: J Neurol. 2024 Feb 28;271(5):2798–809. doi: 10.1007/s00415-024-12233-3 (PMC11055726; doi:10.1007/s00415-024-12233-3)
Supplement: Supplementary file 1 — Supplementary file1 (DOCX 14 KB) [file 415_2024_12233_MOESM1_ESM.docx]

**Supplementary Material 1.**

***Neuropsychological assessment***

Patients completed neuropsychological assessment at each annual assessment including an evaluation of global cognitive functioning, (Montreal Cognitive Assessment (MoCA), working memory (Letter-Number Sequencing (LNS), processing speed (Symbol Digit Modalities Test (SDMT), visuospatial function (Benton Judgment of Line Orientation Test (BJLOT), language abilities (Semantic Verbal Fluency), learning (Immediate recall score of Hopkins Verbal Learning Test-Revised (HVLT-R) and long-term verbal memory (Delayed free recall score of HVLT-R). Raw scores of neuropsychological tests were employed. References of the abovementioned tests are listed below.

**References**

*Montreal Cognitive Assessment*

Nasreddine ZS, Phillips NA, Bédirian V, et al. The Montreal Cognitive Assessment, MoCA: A brief screening tool for mild cognitive impairment. J Am Geriatr Soc. Epub 2005.

*Letter-Number Sequencing*

Egeland J. Measuring working memory with digit span and the letter-number sequencing subtests from the WAIS-IV: Too low manipulation load and risk for underestimating modality effects. Appl Neuropsychol. Epub 2015.

*Symbol Digit Modalities Test*

Smith a. Symbol Digit Modalities Test (SDMT). Neuropsychol Assess. Epub 2004.

*Benton Judgment of Line Orientation Test*

Benton AL, Varney NR, Hamsher K Des. Visuospatial Judgment: A Clinical Test. Arch Neurol. Epub 1978.

*Semantic Verbal Fluency*

Gladsjo JA, Schuman CC, Evans JD, Peavy GM, Miller SW, Heaton RK. Norms for letter and category fluency: Demographic corrections for age, education, and ethnicity. Assessment. Epub 1999.

*Hopkins Verbal Learning Test-Revised*

Hopkins Verbal Learning Test Revised. Definitions. 2020.
