# Supplementary material for: Anatomical correlates of apathy and impulsivity co-occurrence in early Parkinson’s disease
Source: J Neurol. 2024 Feb 28;271(5):2798–809. doi: 10.1007/s00415-024-12233-3 (PMC11055726; doi:10.1007/s00415-024-12233-3)
Supplement: Supplementary file 2 — Supplementary file2 (DOCX 15 KB) [file 415_2024_12233_MOESM2_ESM.docx]

**Supplementary Material 2. Multilevel Models predicting the development of apathy from clinical, neuropsychiatric and socio-demographic variables for ICDs sub-types.**

|  |  | **Apathy** | | |
| --- | --- | --- | --- | --- |
|  |  | *Beta* | *SE* | *p* |
| *Predictors* |  |  |  |  |
| Age | | -0.003 | 0.003 | 0.319 |
| Sex | | -0.044 | 0.038 | 0.248 |
| Level of education | | -0.003 | 0.006 | 0.656 |
| Type of onset | | 0.090 | 0.066 | 0.172 |
| H&Y | | 0.077 | 0.030 | **0.009** |
| UPDRS-III | | 0.005 | 0.001 | **<0.001** |
| LEDD | | -0.000 | 0.000 | 0.850 |
| Levodopa | | 0.139 | 0.049 | **0.005** |
| DAs | | -0.078 | 0.051 | 0.124 |
| Other | | -0.000 | 0.047 | 0.999 |
| Levodopa + Other | | 0.106 | 0.064 | 0.102 |
| Levodopa + DAs | | -0.010 | 0.063 | 0.878 |
| DAs + Other | | 0.050 | 0.057 | 0.380 |
| Levodopa + DAs + Other | | -0.043 | 0.073 | 0.552 |
| Time | | 0.010 | 0.010 | 0.337 |
| Depression | | 0.349 | 0.020 | **<0.001** |
| Anxiety | | 0.111 | 0.019 | **<0.001** |
| Pathological Gambling | | 0.142 | 0.124 | 0.252 |
| Compulsive shopping | | 0.016 | 0.067 | 0.806 |
| Hypersexuality | | -0.015 | 0.064 | 0.809 |
| Compulsive Eating | | 0.148 | 0.048 | **0.002** |
| Hobbyism | | 0.012 | 0.045 | 0.791 |
| Punding | | -0.051 | 0.064 | 0.427 |
| Aimless walkabout | | 0.273 | 0.117 | **0.019** |

ICDs= Impulse control disorders; H&Y= Hoehn and Yahr staging system; UPDRS= Unified Parkinson’s Disease Rating Scale; LEDD= Levodopa Equivalent Daily Dose; DA= Dopamine Agonists
