# Supplementary material for: Anatomical correlates of apathy and impulsivity co-occurrence in early Parkinson’s disease
Source: J Neurol. 2024 Feb 28;271(5):2798–809. doi: 10.1007/s00415-024-12233-3 (PMC11055726; doi:10.1007/s00415-024-12233-3)
Supplement: Supplementary file 3 — Supplementary file3 (DOCX 19 KB) [file 415_2024_12233_MOESM3_ESM.docx]

**Supplementary Material 3. Multilevel Models predicting the performance on LNS from demographic, clinical and neuropsychiatric variables.**

|  |  | **LNS (general ICDs)** | | | | | **LNS (ICDs sub-types)** | | | |
| --- | --- | --- | --- | --- | --- | --- | --- | --- | --- | --- |
|  |  |  | |  | |  | |  |  |  |
|  |  | *Beta* | *SE* | | *p* | | *Beta* | | *SE* | *p* |
| *Predictors* |  |  | |  | |  | |  |  |  |
| Age | | -0.107 | 0.017 | | **<0.001** | | -0.107 | | 0.017 | **<0.001** |
| Sex | | 0.271 | 0.223 | | 0.225 | | 0.291 | | 0.223 | 0.191 |
| Level of education | | 0.159 | 0.036 | | **<0.001** | | 0.158 | | 0.036 | **<0.001** |
| Type of onset | | -0.144 | 0.387 | | 0.710 | | -0.126 | | 0.386 | 0.744 |
| H&Y | | 0.021 | 0.106 | | 0.839 | | 0.019 | | 0.106 | 0.854 |
| UPDRS-III | | -0.009 | 0.005 | | 0.105 | | -0.009 | | 0.005 | 0.089 |
| LEDD | | 0.000 | 0.000 | | 0.618 | | 0.000 | | 0.000 | 0.661 |
| Time | | -0.120 | 0.029 | | **<0.001** | | -0.121 | | 0.029 | **<0.001** |
| Depression | | -0.133 | 0.078 | | 0.086 | | -0.126 | | 0.077 | 0.105 |
| Anxiety | | -0.088 | 0.070 | | 0.207 | | -0.091 | | 0.070 | 0.192 |
| Apathy | | -0.163 | 0.083 | | **0.050** | | -0.113 | | 0.078 | 0.148 |
| ICDs | | -0.313 | 0.079 | | **<0.001** | | - | | - | - |
| Apathy x ICDs | | 0.145 | 0.077 | | 0.061 | | - | | - | - |
| Gambling | | - | - | | - | | 0.171 | | 0.450 | 0.703 |
| Buying | | - | - | | - | | -0.628 | | 0.238 | **0.008** |
| Sex | | - | - | | - | | -0.162 | | 0.236 | 0.491 |
| Eating | | - | - | | - | | -0.184 | | 0.174 | 0.291 |
| Hobbyism | | - | - | | - | | 0.084 | | 0.160 | 0.601 |
| Punding | | - | - | | - | | -0.647 | | 0.227 | **0.004** |
| Walking | | - | - | | - | | -0.218 | | 0.401 | 0.587 |

LNS= Letter Number Sequencing; H&Y= Hoehn and Yahr staging system; UPDRS= Unified Parkinson’s Disease Rating Scale; LEDD= Levodopa Equivalent Daily Dose; ICDs= Impulse control disorders.
