# Supplementary material for: Anatomical correlates of apathy and impulsivity co-occurrence in early Parkinson’s disease
Source: J Neurol. 2024 Feb 28;271(5):2798–809. doi: 10.1007/s00415-024-12233-3 (PMC11055726; doi:10.1007/s00415-024-12233-3)
Supplement: Supplementary file 4 — Supplementary file4 (DOCX 22 KB) [file 415_2024_12233_MOESM4_ESM.docx]

**Supplementary Material 4. Multilevel Models predicting cognition from demographic, clinical and neuropsychiatric variables.**

|  |  | **MoCA** | | | **HVLT – Immediate recall** | | | **HVLT – Delayed recall** | | |
| --- | --- | --- | --- | --- | --- | --- | --- | --- | --- | --- |
|  |  | *Beta* | *SE* | *p* | *Beta* | *SE* | *p* | *Beta* | *SE* | *p* |
| *Predictors* |  |  | | | | | |  |  |  |
| Age | | -0.079 | 0.018 | **<0.001** | -0.193 | 0.033 | **<0.001** | -0.110 | 0.017 | **<0.001** |
| Sex | | 0.723 | 0.232 | **0.002** | 2.217 | 0.424 | **<0.001** | 1.115 | 0.220 | **<0.001** |
| Level of education | | 0.124 | 0.037 | **0.001** | 0.298 | 0.068 | **<0.001** | 0.174 | 0.035 | **<0.001** |
| Type of onset | | -0.351 | 0.402 | 0.383 | -0.374 | 0.734 | 0.610 | 0.219 | 0.381 | 0.567 |
| H&Y | | 0.078 | 0.120 | 0.515 | 0.001 | 0.220 | 0.996 | -0.221 | 0.116 | 0.056 |
| UPDRS-III | | -0.035 | 0.006 | **<0.001** | -0.023 | 0.011 | **0.034** | -0.006 | 0.006 | 0.315 |
| LEDD | | -0.001 | 0.000 | **0.001** | -0.000 | 0.000 | 0.179 | -0.000 | 0.000 | 0.156 |
| Time | | 0.009 | 0.033 | 0.781 | 0.062 | 0.060 | 0.302 | 0.051 | 0.032 | 0.106 |
| Depression | | -0.273 | 0.088 | **0.002** | -0.442 | 0.161 | **0.006** | -0.233 | 0.084 | **0.006** |
| Anxiety | | 0.005 | 0.079 | 0.948 | 0.151 | 0.145 | 0.297 | 0.016 | 0.076 | 0.831 |
| Apathy | | -0.285 | 0.095 | **0.003** | -0.132 | 0.173 | 0.446 | -0.169 | 0.091 | 0.063 |
| ICDs | | -0.037 | 0.090 | 0.678 | -0.198 | 0.164 | 0.226 | -0.133 | 0.086 | 0.123 |
| Apathy x ICDs | | 0.086 | 0.088 | 0.327 | 0.096 | 0.160 | 0.548 | 0.051 | 0.084 | 0.542 |

ICDs= Impulse control disorders; H&Y= Hoehn and Yahr staging system; UPDRS= Unified Parkinson’s Disease Rating Scale; LEDD= Levodopa Equivalent Daily Dose; MoCA= Montreal Cognitive Assessment; HVLT= Hopkins Verbal Learning Test.

|  |  | **BJLOT** | | | **SFT** | | | **SDMT** | | |
| --- | --- | --- | --- | --- | --- | --- | --- | --- | --- | --- |
|  |  | *Beta* | *SE* | *p* | *Beta* | *SE* | *p* | *Beta* | *SE* | *p* |
| *Predictors* |  |  | | | | | |  |  |  |
| Age | | -0.040 | 0.014 | **0.004** | -0.322 | 0.076 | **<0.001** | -0.501 | 0.064 | **<0.001** |
| Sex | | -1.063 | 0.176 | **<0.001** | 6.975 | 0.978 | **<0.001** | 3.218 | 0.822 | **<0.001** |
| Level of education | | 0.147 | 0.028 | **<0.001** | 0.808 | 0.157 | **<0.001** | 0.674 | 0.132 | **<0.001** |
| Type of onset | | -0.134 | 0.304 | 0.659 | -1.108 | 1.699 | 0.515 | 0.540 | 1.427 | 0.705 |
| H&Y | | 0.040 | 0.096 | 0.678 | 0.023 | 0.392 | 0.954 | 0.415 | 0.369 | 0.262 |
| UPDRS-III | | -0.015 | 0.005 | **0.001** | -0.059 | 0.020 | **0.003** | -0.148 | 0.019 | **<0.001** |
| LEDD | | -0.000 | 0.000 | 0.217 | -0.000 | 0.001 | 0.467 | -0.000 | 0.001 | 0.533 |
| Time | | -0.009 | 0.026 | 0.718 | -0.136 | 0.107 | 0.204 | -0.285 | 0.101 | **0.005** |
| Depression | | -0.030 | 0.070 | 0.665 | 0.227 | 0.286 | 0.428 | -0.191 | 0.270 | 0.480 |
| Anxiety | | -0.121 | 0.063 | 0.057 | -0.241 | 0.258 | 0.351 | -0.636 | 0.243 | **0.009** |
| Apathy | | -0.137 | 0.076 | 0.072 | -0.414 | 0.308 | 0.180 | -1.061 | 0.290 | **<0.001** |
| ICDs | | 0.023 | 0.072 | 0.749 | -0.080 | 0.292 | 0.784 | -0.450 | 0.275 | 0.102 |
| Apathy x ICDs | | 0.003 | 0.071 | 0.964 | -0.179 | 0.283 | 0.527 | 0.388 | 0.267 | 0.146 |

ICDs= Impulse control disorders; H&Y= Hoehn and Yahr staging system; UPDRS= Unified Parkinson’s Disease Rating Scale; LEDD= Levodopa Equivalent Daily Dose; BJLOT= Benton Judgment of Lines Orientation; SFT= Semantic Fluency Test; SDMT= Symbol Digit Modalities Test.
